# The neurobiology of irritable bowel syndrome

**DOI:** 10.1038/s41380-023-01972-w

**Published:** 2023-02-02

**Authors:** Emeran A. Mayer, Hyo Jin Ryu, Ravi R. Bhatt

**Affiliations:** 1 G. Oppenheimer Center for Neurobiology of Stress and Resilience, Departments of Medicine, Psychiatry and Physiology, David Geffen School of Medicine at UCLA, Los Angeles, CA USA; 2 A.T. Still University School of Osteopathic Medicine in Arizona, Meza, AZ USA; 3 Imaging Genetics Center, Mark and Mary Stevens Neuroimaging and Informatics Institute, Keck School of Medicine at USC, University of Southern California, Los Angeles, CA USA

**Keywords:** Neuroscience, Genetics, Diseases

## Abstract

Irritable bowel syndrome (IBS) is the most prevalent disorder of brain-gut interactions that affects between 5 and 10% of the general population worldwide. The current symptom criteria restrict the diagnosis to recurrent abdominal pain associated with altered bowel habits, but the majority of patients also report non-painful abdominal discomfort, associated psychiatric conditions (anxiety and depression), as well as other visceral and somatic pain-related symptoms. For decades, IBS was considered an intestinal motility disorder, and more recently a gut disorder. However, based on an extensive body of reported information about central, peripheral mechanisms and genetic factors involved in the pathophysiology of IBS symptoms, a comprehensive disease model of brain-gut-microbiome interactions has emerged, which can explain altered bowel habits, chronic abdominal pain, and psychiatric comorbidities. In this review, we will first describe novel insights into several key components of brain-gut microbiome interactions, starting with reported alterations in the gut connectome and enteric nervous system, and a list of distinct functional and structural brain signatures, and comparing them to the proposed brain alterations in anxiety disorders. We will then point out the emerging correlations between the brain networks with the genomic, gastrointestinal, immune, and gut microbiome-related parameters. We will incorporate this new information into a systems-based disease model of IBS. Finally, we will discuss the implications of such a model for the improved understanding of the disorder and the development of more effective treatment approaches in the future.

## Introduction

IBS is one of the most common disorders of brain-gut interaction globally, with prevalence rates between 1.1 and 45% worldwide, and between 5 and 10% for most Western countries and China [1]. In contrast to many chronic non-communicable diseases, such as metabolic, neurological, cardiovascular and some forms of cancer, there has been no progressive increase in prevalence during the past 75 years, even though prevalence numbers have been fluctuating due to the periodic changes in official symptom criteria. Based on questionnaire data, women are 1.5–3.0 times more likely to have IBS, reflecting a prevalence in women of 14% and in men of 8.9% [2, 3]. However, based on healthcare system utilization, women are up to 2–2.5 times more likely to see a healthcare provider for their symptoms [4]. Based on the current symptom criteria [5], IBS is defined by chronically recurring abdominal pain associated with altered bowel habits in the absence of detectable organic disease. IBS symptoms can be debilitating in a small number of patients, but are mild to moderate in the majority of affected individuals [6]. Based on this definition, other frequently associated somatic or visceral pain and discomfort, as well as anxiety and depression are considered so called comorbid conditions.

The gut-restricted definition of the Rome criteria overlooks the fact that a large number of individuals who meet diagnostic criteria for an anxiety or depressive disorder have IBS and vice versa [7–10], and a majority of IBS patients show elevated levels of trait anxiety and neuroticism [10–13], or meet diagnostic criteria for an anxiety disorder [14]. Currently, the commonly associated psychiatric and somatic symptoms are generally referred to as comorbidities, separate from the primary GI diagnosis [15] and not present in all patients. However, detailed patient histories, frequently reveal symptoms of abdominal discomfort, anxiety and behavioral disturbances starting in early childhood in a majority of patients, and a large recent genetic epidemiological study has provided an intriguing explanation for the co-occurrence of abdominal and psychiatric symptoms in IBS patients on the basis of several shared single nucleotide polymorphisms (see paragraph *IBS related genes shared with anxiety disorders* below) [8]. These new findings are consistent with genetic vulnerabilities affecting both the central and the enteric nervous system (ENS), and argue against the long held linear pathophysiological concepts that emotional factors may cause IBS symptoms, or that chronic IBS gut symptoms lead to anxiety and depression Box 1.

Much of research and drug development in IBS patients has been based on descriptive and symptomatic features, rather than on biology-based disease definitions. These definitions suggest a core abnormality shared by all IBS patients (chronic, recurrent abdominal pain) as well as heterogeneity based on self reports of predominant bowel habit. However, a comprehensive identification of distinct biology-based subgroups of patients including those based on sex, with different underlying pathophysiological components and differential responsiveness to specific therapies, has not been achieved. Subtypes based on bowel habits are generally based on subjective reports of altered bowel habits, without consistent correlates in intestinal transit times, altered regional motility patterns or altered fluid and electrolyte handling by the gut [16]. Even though some of the most commonly used pharmacological and behavioral therapies are targeted at the level of the brain (low dose tricyclic antidepressants [17], serotonin reuptake inhibitors [18], cognitive behavioral therapies [19, 20], gut directed hypnosis, stress management [21]), research and drug development efforts are still predominantly focused on single, usually peripheral targets identified in preclinical models [16].

Based on such studies and on clinical reports from small samples, an astonishing list of biological abnormalities at various levels of the brain gut axis have been reported in the last 30 years and proposed as potential biomarkers or pathophysiological factors [2]: smooth muscle cells [22, 23], the gut epithelium [24]; bile acids [25–28]; immune system activation [29, 30]; neuroendocrine mechanisms [31]; brain structure and function [32, 33]; stress responsiveness [34]; affective [35, 36], cognitive [37–40], pain modulation [41, 42], gene polymorphisms [8]; and most recently the gut microbiome [43–47]. In addition, there has been a wealth of comprehensive data and clinical reports demonstrating a strong relationship between psychosocial factors and IBS symptoms [48]. However, despite the emergent discoveries about possible peripheral [29, 30] and central [32, 33, 35, 49, 50] components in IBS pathophysiology, the development of animal models with high face and construct validity [51], the reproduction of visceral hypersensitivity and IBS-relevant features after transplantation of human biospecimen into rodent models, and the recent acceptance of a brain-gut model of IBS [52], the controversy on the primary role of the nervous system versus peripheral factors still persists in the field [33, 53].

**Fig. 1:**
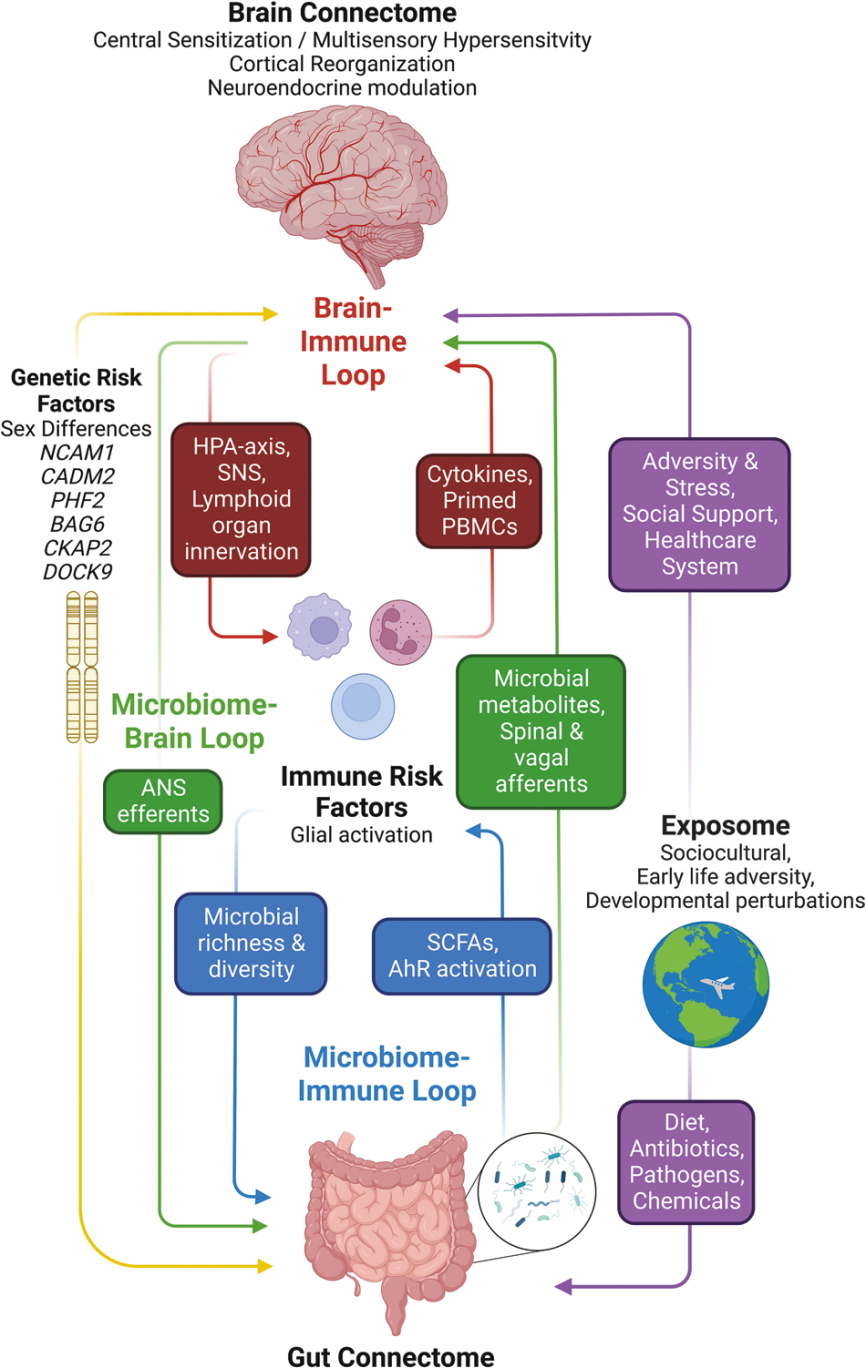
The brain-gut-microbiome system. The brain connectome, gut connectome and gut microbiome communicate in a bidirectional way. The response characteristics of the system are determined by vulnerability genes interacting with different influences from the exposome. The different loops use neural, endocrine, paracrine and immune signaling mechanisms. Perturbations (stressors) of the different nodes of the system (brain, gut, immune, microbiota) result in non-linear effects and alterations in response characteristics manifesting as psychiatric and/or gut symptoms. ANS autonomic nervous system, SNS sympathetic nervous system, PBMCs peripheral blood mononuclear cells, SCFAs short chain fatty acids, AhR aryl hydrocarbon receptor.

In this review, we will discuss the evidence supporting an integrative brain gut microbiome (BGM) model (Fig. 1) which incorporates a large body of evidence from studies on peripheral and central neurobiological disease mechanisms, brain and gut targeted influences of the exposome, and results from recently reported large scale genetic analyses with relevance for neuronal dysfunction of the CNS (central nervous system) and ENS (enteric nervous system). This systems biological model is consistent with the frequent comorbidity of IBS with other so-called functional GI disorders, and with other chronic pain and psychiatric disorders, in particular with anxiety. We will use this model to discuss the implications for the pathophysiology of IBS, its association with psychiatric symptoms, and the development of more effective treatment approaches in the future.

Box 1 Brain-gut-system abnormalities reported in IBS **Gastrointestinal**

 Altered intestinal motility and transit time

 Altered fluid secretion/absorption

 Hypersensitivity of visceral afferents

 Altered mucus layer

**Gut microenvironment**

 Altered microbiome composition

 Altered fecal bile acid profile

 Increased intestinal barrier permeability

**Neurological**

 Structural and functional brain alterations

 Alterations in brain receptors for cortical corticotropin release factor (CRF), neurokinin-1 (NRK-1), and cannabinoid-1 receptor systems

**Genetic**

 Female sex

 Gene polymorphisms

 *CADM2*

 *BAG6*

 *PHF2, FAM120AOS*

 *NCAM1*

 *CKAP2, TPTE2P3*

 *DOCK9*

## The brain-gut-microbiome system

### The enteric nervous system and gut connectome

The ENS is a vast network of different types of intrinsic enteric neurons and glia which are “sandwiched” between the mucosa, and the circular and longitudinal muscle layers of the gut, containing motor neurons, intrinsic primary afferent neurons, and interneurons. Nearly every neurotransmitter class found in the CNS is present in the ENS [54]. These neurons are organized into two interconnected networks, the myenteric and submucosal plexus, which regulate motility and secretion respectively in a coordinated fashion [55]. Different classes of neurons are chemically coded by different combinations of neurotransmitters and modulators, many of which are also found in the CNS [56].

**Fig. 2:**
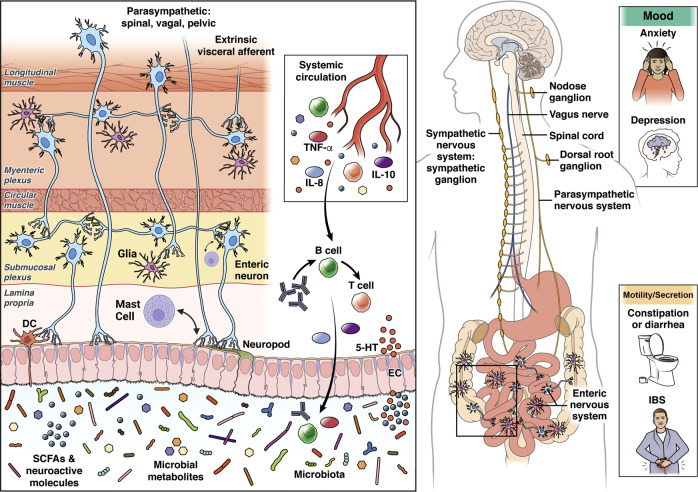
Bidirectional interactions of the gut microbiome with the Enteric Nervous System, the enteroendocrine system, the gut-associated immune system, and the brain. Alterations in these interactions can present as psychiatric and/or IBS symptoms. Modified with permission from [79].

Within the gut, the ENS is closely connected with the gut-based immune system, endocrine system, glial and epithelial cells, making up the gut connectome [57] (Fig. 2). The term connectome reflects close proximity, connectivity, and functional interactions between many cell types and functions in the gut that interact with ENS and CNS.

Beyond the gut, the ENS is connected with the spinal cord, brainstem, and brain via primary spinal and vagal afferents, and postganglionic sympathetic and vagal efferent fibers [58, 59]. Although the ENS is capable of regulating all GI functions without input from the CNS, the CNS (brain and spinal cord) has strong modulatory functions in regulating intestinal behaviors [60] in accordance with the overall state of the organisms and homeostatic perturbations [53].

Even though the ENS is often being referred to as the “second brain” [61], evolutionarily speaking, the ENS can be traced back to the *cnidaria* phylum and epitomized by the *hydra* genus 650 million years ago [62]. Historically, it has been classified as a nerve net, but evidence has shown specialized neurons with neurotransmitters such as serotonin, catecholamines, and neuropeptides are also involved [63, 64]. In the *hydra*, the main function of the ENS is peristalsis, mixing movements and expulsion in addition to avoidance behaviors, [62]. The process of cephalization and the development of *bilateria* (i.e., organisms through evolution with a head/tail [anterior/posterior axis] and belly/back [dorsal/ventral axis]) led to the development of more complex neuronal systems, most notably the CNS around a central region and highly developed brains. Thus from an evolutionary standpoint, the ENS can be considered “the first brain” [56, 62].

### ENS related genes

A recent profiling of the human ENS at single-cell resolution highlighted important genes related to neuropathic, inflammatory, and extraintestinal diseases [65]. Overlapping with the largest GWAS of IBS to date [8], *CADM2*, encoding the cell-adhesion molecule, was highly expressed in myenteric but not mucosal glia [65]. The known functions of myenteric glia include modulating myenteric neuron activity, regulating oxidative stress and neuroinflammation, providing trophic support, gliogenesis, and neurogenesis [66]. *CADM2* encodes a member of synaptic cell adhesion molecules (SynCAMs) involved in synaptic organization and signaling [67], and cell adhesion-mediated mechanisms underlying the communication between glia and neurons in the ENS are important in understanding of ENS function in health and disease. For example, perturbed communication between enteric glia and neurons may play a role in dysfunctional ENS circuits in IBS [66]. The mechanisms underlying neuronal-glia signaling of the ENS in the context of gastrointestinal disorders, IBS, and visceral pain has recently been extensively reviewed [66, 68]. It is worth noting that *CADM2* has been implicated in a wide range of psychological and neurological traits often observed in IBS patient including, but not limited to psycho-behavioral traits, risk-taking behavior, nervousness-like traits, and neurodevelopmental disorders (e.g., intellectual disability and autism spectrum disorder) [69]. Moreover, SynCAMs have a large role in synaptogenesis, axon guidance, and synaptic plasticity at a basic neurodevelopmental level which has the potential to affect a variety of disorders [70].

Similarly, *NCAM1* is another gene found in the largest GWAS to date and has been implicated in the development of the ENS. In a similar manner to *CADM2*, *NCAM1* has been shown to play a role in the ENS regarding cell migration, axon growth, neuronal plasticity and fasciculation [71], but has not been as thoroughly investigated as *CADM2*. A recent cross-tissue atlas applied single-nucleus RNA sequencing from eight healthy human organs showed that a cluster of genes including *NCAM1* and *CADM2* were involved particularly with cognitive/psychiatric symptoms including general cognitive ability, risk-taking behavior, intelligence, and neuroticism [72]. Even though the study did not contain tissue samples from the intestinal regions of the ENS, these genes involved in cognitive/psychiatric functions were highly expressed in Schwann cells in the esophagus mucosa, and interstitial cells of Cajal (ICCs) and neurons in the esophagus muscularis [72].

### The gut microbiome

The term gut microbiome refers to the 40 trillion microbial organisms (bacteria, fungi, and archae) and their millions of genes that live throughout the gastrointestinal tract, from the oral cavity to the rectum, with the highest concentration and diversity in the large bowel [73]. The symbiotic interactions of the 3 groups of microorganisms within the microbiome, and with the extensive gut virome are incompletely understood [74, 75]. The characterization of these microorganisms in IBS to date is primarily based on identification of relative abundances and diversity using 16S rRNA sequencing techniques with limited resolution beyond the species level. We refer to several recent review articles on this topic [76, 77]. The extensive literature reveals inconsistent findings and a causative relationship of specific microorganisms with IBS symptoms has not been demonstrated. However, both preclinical and some clinical studies have demonstrated a significant effect of psychosocial stress on the relative abundance of gut microbes which is mediated both by stress-induced alterations in regional transit and secretion, and by direct effects of norepinephrine and possibly other signaling molecules released from gut cells on gut microbial gene expression and virulence [78], suggesting the possibility that the microbiome in subgroups of IBS patients with greater stress reactivity may contribute to certain symptoms [79].

### Brain Connectome alterations in IBS

A growing body of research paired with clinical observations supports a critical role of the brain in the generation and maintenance of IBS symptoms. Regardless of primary symptom triggers, the brain is ultimately responsible for constructing and generating the conscious perception of abdominal pain, discomfort, and anxiety based on sensory input from the gut. Stressful and traumatic events during early life increase chances of developing IBS, and psychosocial stressors in adulthood play a crucial role during the first onset, symptom flare, and perceived severity of the symptoms [80]; centrally targeted pharmacological treatments and cognitive behavioral strategies have been some of the most effective IBS treatment strategies [3, 16, 81].

**Fig. 3:**
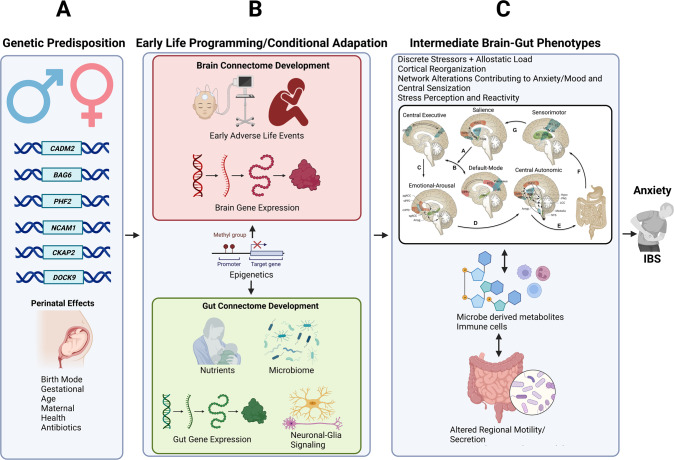
Programming of the brain and gut connectome based on shared vulnerability genes and environmental influences. **a** Vulnerability genes and prenatal influences (including maternal health, nutrition, and stress level) on BGM system development. **b** the brain and gut transcriptome is influenced by mode of delivery, early adversity, and early nutrition, leading to the development of distinct intermediate brain gut phenotypes **c** which shape the adult response to influences from the exposome (diet, psychosocial stress).

Specific brain functions such as sensory processing and modulation, emotion regulation, or cognition are the result of dynamic interactions of distributed brain areas operating in large-scale networks. As summarized in Fig. 3C and Table 1, these central networks and their properties have been assessed by neuroanatomical and neurophysiological studies in animals [51], as well as by a wealth of studies using different structural and functional brain imaging techniques and analyses in humans [82–86].

**Table 1:** Brain network alterations in IBS.

**Default Mode Network**	**Brain Regions**	Medial prefrontal cortex (mPFC); posterior cingulate or retrosplenial cortex; precuneus; inferior parietal cortex; lateral temporal cortex; hippocampal formation
	**Function**	• self-awareness processing, episodic memory, monitoring internal thoughts, external goals, and future planning [182–185]
	**Alterations in IBS**	• altered functional connectivity and topological reorganization in various regions, consistent with dysregulation in chronic visceral pain [111]. Lower morphological integrity, resting-state, and anatomical connectivity predicts symptom exacerbation over time in women with IBS [109]
	**Shared alterations in anxiety disorders**	• Reduced resting-state functional connectivity across anxiety disorders [186–188]. • Lower volume in mPFC and hippocampus [189]
	**Treatment response**	• Responders to CBT have reduced functional connectivity between the brainstem and lateral temporal cortices, sensorimotor network regions and lateral temporal cortices, and amygdala and lateral temporal cortices [177]. • Responders to hypnotherapy show a reduced activation of ventromedial PFC to rectal distension [172]. • Increased coherence of DMN in response to rectal lidocaine, which is associated with decreased perceived pain [113]
	**Sex** **Difference**	• N/A
**Sensorimotor** **Network**	**Brain Regions**	Thalamus; basal ganglia (BG); sensorimotor cortex; posterior operculum/INS; area 24 of the cingulate cortex.
	**Function**	Processing and modulation of visceral and somatic sensory information [190]
	**Alterations in IBS**	• Increased frequency power of spontaneous brain oscillations [100] • Widespread microstructural white matter changes [114, 191] • Female IBS greater volume and cortical thickness, correlated with symptom severity [115, 117] • Greater gray matter in pINS, correlated with symptom duration [116] • ACC and thalamus are hubs in structural network analysis [117] • Greater betweenness centrality of cingulate gyrus and thalamus [117] • Lower gray matter volume in thalamus and basal ganglia in adolescent girls. Thalamus volume is inversely associated with heat pain threshold. Greater functional connectivity between the caudate nucleus and precentral gyrus [108]. • Greater cortical thickness and volume in the sensorimotor network and basal ganglia respectively. Lower cortical thickness in the posterior insula. Pain intensity during rectal distention associated with primary somatosensory cortex thickness and pain threshold is associated with nucleus accumbens volume [192]. • Greater sensorimotor-salience resting-state and anatomical connectivity predicts symptom exacerbation over time in women with IBS [109]
	**Shared alterations by anxiety disorders**	• Increased top-down sensorimotor resting-state effective connectivity in sensorimotor cortices [193] • Increased sensorimotor connectivity associated with panic-related symptoms [187, 188]. • Lower thalamus volume [194]
	**Treatment Response**	• Responders to CBT have decreased functional connectivity between the pINS and brainstem, salience network, and DMN [177]. • Responders to hypnotherapy show a reduced activation of pINS to rectal distension [172]. • 5-HT3 receptor antagonism is associated with decreased activity in the basal ganglia in response to rectosigmoid stimulation [195]. • NK-1R antagonism is associated with decreased activity in pINS in response to visceral distension [196]
	**Sex Difference**	• Females with IBS have lower fractional anisotropy (FA) by diffusion MRI in the thalamus and basal ganglia, and greater mean diffusivity in the thalamus and basal ganglia [191]. • Females with IBS have lower FA in the thalamus, primary sensory cortex, and lower MD in globus pallidus [114]
**Salience** **Network**	**Brain Regions**	mPFC, OFC, mid ACC; aINS; amygdala
	**Function**	• Detection of behaviorally relevant stimuli, response to experience or expectation of any interoceptive and exteroceptive stimulus threatening homeostasis [82, 103, 197] • Coordination of the appropriate attentional, behavioral, affective, and autonomic nervous system responses to such stimuli [82, 197]
	**Alterations in IBS**	• Greater engagement of aINS and mACC in response to actual and expected rectal distension [32]. • Increased affective, central, emotional arousal processes as well as enhanced visceral stimulus perception [104–106]. • Alterations in the activity and connectivity of aINS in females both during the resting-state [99, 100] and abdominal pain threat [198] • Lower NKR-1 receptor availability in the aMCC [107] • Lower gray matter volume in the aMCC in adolescent girls [108]. • Greater salience-sensorimotor connectivity predicts symptom exacerbation in women with IBS [109]
	**Shared alterations by anxiety disorders**	• Greater resting-state salience network functional connectivity and reactivity in generalized and social anxiety disorders [187, 188, 199] • Lower volume in the aMCC [189].
	**Treatment Response**	• Responders to CBT have reduced functional connectivity between the aINS and dorsal ACC, aINS and sensorimotor network, and aINS and DMN [177]. • Responders to hypnotherapy show reduced activation of aINS, aMCC to rectal distension. Reduced activation of the aINS was associated with reduced visceral sensitivity [172]. • NK1R antagonist is associated with decreased activity in the aMCC in response to visceral distension [196]
	**Sex Difference**	• Females had greater negative connectivity of aINS to mPFC [99].
**Emotional** **Arousal** **Network**	**Brain Regions**	mPFC, ventrolateral prefrontal cortex (vlPFC), amygdala, hippocampus, hypothalamus, posterior, subgenual cingulate cortex (sgACC), and locus coeruleus (LC).
	**Function**	○ Activated by perceived or real disruption in homeostasis [200] ○ Generation of rapid feedback inhibition of amygdala, thereby limiting the magnitude and duration of network activity and related activity in the central autonomic network [201, 202]
	**Alterations in IBS**	• Decrease in inhibitory feedback loop [94, 104, 170]; also seen in healthy controls whose central serotonin levels were lowered by acute tryptophan depletion [203]. • Increased responsiveness to both expected and delivered visceral stimuli in females [139, 204, 205]. • More consistent activation in response to controlled rectal distension [95]. • reactivity associated with serotonin (5-HT)-related gene polymorphisms [206]. • Functional alterations are accompanied by structural brain alterations [117]
	**Shared alterations by anxiety disorders**	• Lower amygdala, striatum, hippocampus, and hypothalamus volume [194] • Greater functional connectivity of emotion regulating regions to the sensorimotor and salience networks [194]
	**Treatment Response**	• Responders to CBT have reduced functional connectivity between the amygdala and the DMN [177]. • Responders to hypnotherapy show a reduced activation of aINS, sgACC, hippocampus to rectal distension. Reduced hippocampus activation was associated with reduced GI symptoms [172]. • CRF-R1 receptor antagonism induces greater BOLD reduction in the hypothalamus of IBS patients compared to controls, moderated by anxiety [170]. • 5-HT3 receptor antagonism is associated with decreased activity in the sgACC, amygdala and hippocampus in response to rectosigmoid stimulation [195]. • NK-R antagonism is associated with decreased activity in the amygdala and hippocampus in response to visceral distension [196]
	**Sex Difference**	• Greater emotional-arousal network reactivity and altered connectivity in female IBS [91]. • Greater emotional-arousal network reactivity to specific stimuli (faces depicting fear and anger) in male IBS [94]. • Females with IBS have lower cortical thickness in the sgACC compared to males with IBS [115]
**Central Autonomic** **Network**	**Brain Regions**	Control centers in the pontine-medulla (including PAG and hypothalamus), LC, the central nucleus of the amygdala, and several cortical regions (including the anterior INS, sgACC, ACC, prefrontal, and motor regions)
	**Function**	• Central control and modulation of the autonomic nervous system [118, 119] • Regulation of respiratory, cardiovascular, endocrine, and digestive activities during cognitive, affective, and motor tasks and sensations [119]
	**Alterations in IBS**	• Alterations in CRF-R1 [170, 171] and norepinephrine–adrenergic receptor signaling system [205]. • Reduced inhibition of dorsal brainstem regions during anticipation associated with greater activity in the orbitofrontal cortex and sgACC during rectal distension [205].
	**Shared alterations by anxiety disorders**	• Greater volume of the ventral diencephalon in males with generalized anxiety disorder [207]. • Altered functional connectivity between the amygdala and frontal regions [188]
	**Treatment Response**	• Greater connectivity between the central autonomic network and emotional arousal network in individuals who are responders to CBT treatment [177]. • 5-HT3 receptor antagonism is associated with decreased activity in the hypothalamus and amygdala in response to rectosigmoid stimulation [195]
	**Sex Difference**	• Greater activation of dorsolateral PFC INS and dorsal pons/PAG in response to visceral stimulus in male IBS [91]. • Greater activation of ventromedial PFC, right ACC and left amygdala in response to visceral stimulus in female IBS [91]. • Females have greater mean diffusivity via diffusion imaging in the brainstem [191].
**Central** **Executive** **Network**	**Brain Regions**	Lateral PFC and posterior parietal cortices
	**Function**	• Activated during tasks involving executive functions such as attention, working memory, planning and response selection [208–210] • Often co-activated with regions of the SN, as the brain attempts to focus its limited processing capacity to only salient information via attention, working memory, planning and response selection [211]
	**Alterations in IBS**	• Deficient activation of inhibitory cortical regions involved in down-regulation of pain and emotion as well as attention during expectation and experience of aversive gastrointestinal stimuli [95]. • Selective recall of negative and gastrointestinal sensation words, as well as selective attention to threat-related stimuli [212–215]. • Reduced effective connectivity during repeated exposure to the anticipation and experience of a threatening gastrointestinal stimulus, which was linked to a reduction in IBS hypersensitivity [216]. • Altered error feedback mechanisms linked to decreased dorsolateral PFC activity in Japanese IBS patients [37]. • Strong negative association between the cortical thickness and gray matter density of the dorsolateral PFC and pain catastrophizing [217, 218]. • Altered prepulse inhibition (a process by which an organism can filter the flow of information from its internal and external environments) [101]. • Lower gray matter volume in the dorsolateral PFC in adolescent girls [108]. • Lower cortical thickness in the superior frontal gyrus/sulcus in patients with IBS [192]. • Lower morphological integrity, anatomical and resting-state connectivity in the dorsolateral PFC predicts symptom exacerbation over time in women with IBS [109]
	**Shared alterations by anxiety disorders**	• Lower functional coherence in the dorsolateral PFC and inferior parietal gyrus in social anxiety disorder [219]. • Reduced dorsolateral PFC activity in response to passive, congruency, emotion modulation and memory tasks [189]. • Lower dorsolateral lPFC resting-state functional connectivity [189]
	**Treatment Response**	• N/A
	**Sex Difference**	• In response to rectal balloon dilation (evoked pain paradigm), males with IBS had greater activation in the dlPFC [220].

In humans, several types of networks have been reported [33] (summarized in Table 1): functional brain networks based on evoked responses [87] or intrinsic connectivity of the brain during rest [82, 83]; structural networks based on gray matter parameters [88] and white matter properties; and anatomical networks based on white matter connectivities [89]. Both evoked and resting state studies performed in patients with IBS have demonstrated abnormalities in regions and task-related networks linked to salience detection [90, 91], emotional arousal [92–95], central autonomic control [38, 96–98], central executive control [90, 94, 99], and sensorimotor processing [38, 100, 101]. IBS-related alterations in these networks have provided plausible neurobiological substrates for several information-processing abnormalities reported in patients with IBS, such as stress hyperresponsiveness, biased threat appraisal, expectancy of outcomes, cognitive inflexibility, autonomic hyperarousal (emotional arousal and central autonomic networks), symptom-focused attention (central executive network) [33, 53] and cognitive inflexibility (central executive network). Supporting the concept of shared pathophysiological factors (so called p-factors), several reported brain network alterations have also been described in other chronic pain conditions [102] and in anxiety disorders (see Table 1).

### The Salience Network

The salience network (SN) is integral in mediating the switching of activation between the default mode network (DMN) and central executive network, coordinating and adjusting physiologic/behavioral responses to internal and environmental perturbations of homeostasis [103]. Visceral inputs to the affective-motivational component of the SN converge onto the anterior insula coordinating response selection and conflict monitoring with the dACC [103]. Controlled rectal distention in IBS subjects has been shown consistently to result in increased engagement of the core hubs of the SN which are associated with increased affective, emotional, and arousal processes [104–106]. Reduced neurokinin-1 receptor (NK-1R) availability in the dACC, reflecting NK-1R endocytosis in response to substance P release, was found to be associated with duration of IBS symptoms [107]. Increased substance P release is thought to result from noxious visceral stimuli and increased engagement of endogenous pain or stress inhibition systems [107]. In adolescent girls with IBS, lower gray matter volume of the dACC has been observed [108], and greater salience-sensorimotor connectivity quantified by multiple neuroimaging techniques predicts a lack of symptom alleviation over 3–12 months in patients with IBS [109].

### The default mode network (DMN)

The DMN’s role in pain perception is known to act as an opposite manner to the SN, such that the DMN is suppressed when attention is placed on present sensory stimuli, and is activated when attention is engaged with thoughts away from present sensory stimuli and engaged in mind wandering (i.e., thoughts unrelated to the present sensory environment) [110]. Studies in chronic pain subjects have shown altered functional connectivity and topological reorganization in various regions, consistent with DMN dysregulation [111]. Overall neuroimaging research suggests decreased activity of the DMN in patients with IBS [112]. Lower integrity of anatomical connectivity and resting-state functional connectivity, and lower morphological integrity within the DMN (between the aMPFC and PCC) were found to be predictive of sustained IBS symptom severity over 3–12 months [109]. Rectal lidocaine administration in IBS subjects was associated with decreased pain perception and with increased coherence in the DMN [113], supporting an involvement of the DMN in visceral hypersensitivity in patients with IBS.

### The Sensorimotor Network

Similar to other chronic pain disorders, imaging studies in IBS subjects have shown alterations of the sensorimotor network (SMN), consistent with alterations in central processing and modulation of viscerosensory and somatosensory information [32, 100, 109, 114–117]. This network consists of the primary motor cortex, area 24 of the cingulate cortex, premotor cortex, supplementary motor area (SMA), posterior operculum/insula, as well as primary and sensory cortices in the parietal lobe. In addition, lower gray matter volume in the basal ganglia and thalamus as well as greater functional connectivity within the SMN have been observed in young children with chronic pain [108]. Greater intrinsic functional connectivity in adults, greater cortical thickness of the posterior insula positively associated with symptom duration, and increasing functional coupling of area 24 and the thalamus, and greater SMN connectivity to the SN predicting sustained symptoms over 3–12 months [109]. When viewed together, current evidence suggests patients with IBS have functional, morphological, and microstructural SMN alteration, which are likely to play a role in the increased perception of both visceral and somatic stimuli.

### The central autonomic network

The central autonomic network (CAN) regulates visceromotor, neuroendocrine, pain, and behavioral responses essential for survival [118]. Afferents project through the spinal cord and eventually arrive at the main homeostatic processing sites in the brainstem/central autonomic network (including hypothalamus, amygdala, and PAG), and higher cortical processing and modulatory regions [119]. Historically it has been difficult to non-invasively study the brain stem nuclei in humans due to the limited spatial resolution of neuroimaging methods, but new imaging protocols with a resolution of 1mm^3^ and below are allowing new insights [120].

The CAN is closely connected by vagal and sympathetic efferent projections with the ENS, and afferents from the ENS send viscerosensory signals back to the brain. The hubs of the SN also participate in autonomic control via descending projections to the amygdala (tagging emotional valence and engaging autonomic survival responses to behaviorally relevant stimuli), hypothalamus (regulating homeostasis and a pattern generator for the stress response) and brainstem structures including the periaqueductal gray (PAG) and locus coeruleus (LC). The PAG is a key structure for integrating autonomic, pain modulatory/analgesic, and motor responses to stress [121], and the LC-norepinephrine system plays a central role in behavioral arousal and stress responses [122–124].

When viewed together, based on a large number of structural, and functional (resting state and evoked) studies, IBS patients show alterations in several brain networks related to salience assessment, attention, stress perception and responsiveness, and sensory processing. The responsiveness and connectivity of these networks are modulated by several vulnerability genes, which are shared both with ENS genes, and with genes identified in anxiety disorders. Based on these findings, we hypothesize that perturbations of homeostasis arising from the exposome, in the form of psychosocial and gut-targeted stressors interact with genetic factors to a spectrum of clinical phenotypes, ranging from gut symptoms to anxiety.

### IBS-related genes shared with anxiety disorders

Prior to the availability of biobank scale data, many candidate gene studies uncovered potential pathways underlying IBS symptoms. These pathways have been extensively reviewed and include the serotonin pathway, *SCN5A*, and intestinal channelopathy, and sucrase-isomaltase malabsorption [125]. As serotonin is secreted from enteroendocrine cells and activates enteric sensory and motor neurons, expression level alterations in serotonin receptors and transporters are likely to play a potential role in visceral hypersensitivity, pain, intestinal motility, and secretion. *SCN5A* encodes the voltage-gated sodium Na_v_1.5 channel present on interstitial cells of Cajal (ICCs) in the ENS [31, 126]. Genetic mutations on this gene have shown to impair peristalsis and cause constipation, even though slow transit constipation is an uncommon finding in IBS-C [127]. Lastly, two faulty copies of the *SI* gene result in reduced disaccharide activity responsible for degradation of sucrose and starch, resulting in diarrhea and gas production in the large intestine from bacterial fermentation and is termed congenital sucrase-isomaltase deficiency (CSID), and should not be considered as IBS [125]. Even though these findings have established causal relationships between specific genetic abnormalities and non-specific IBS-like GI symptoms in a small number of affected individuals, it is highly unlikely that they play an important role in the great majority of patients.

Recently, the largest genome wide association study with 53,000 cases of IBS across multiple cohorts was completed [8]. In this study, the strongest risk factors for IBS included long-term or recurring antibiotic exposure in childhood, somatic pain conditions (back pain, limb pain, headaches), psychiatric conditions (anxiety, depression, excessive worrying) and fatigue. The genes included *CADM2*, *BAG6*, *PHF2/FAM120AOS*, *NCAM1*, *CKAP2*/*TPTE2P3*, and *DOCK9*. Four of the six loci are highly implicated in anxiety/mood disorders and there was a strong genome-wide genetic correlation of IBS with anxiety, neuroticism, depression, insomnia, and schizophrenia. Moreover, the high genetic correlations persisted after taking into account individuals with phenotypic overlap, suggesting common etiological pathways between IBS and anxiety/mood disorders. Implication of the central nervous system was further suggested by the finding that the six identified loci regulate gene expression in many genes primarily expressed in the brain. As already mentioned under ENS above, the genes *NCAM1* and *CADM2* were two genes which regulate neural circuit formation and influence changes in white matter microstructure in IBS and mood disorders [128–130]. Specifically, they regulate synaptic cell adhesion molecules, which are present in dorsal root ganglia sensory neurons throughout development, mediate adhesion of sensory axons, and induce neurite outgrowth [130]. Mechanisms relating to brain development were further implicated by the genes *PHF2* (i.e., proper expansion of neural progenitors) and *DOCK9* (i.e., dendritic development of the hippocampus), but have not yet been studied in patients with IBS [131–133].

Importantly, the heritability of IBS was estimated to be a modest 5.8%, suggesting that perturbation of the brain-gut axis by environmental factors arising from the exposome such as early adversity, psychosocial stress, learned behaviors, diet, and possibly dysbiosis play a prominent role.

Considering these new genetic findings and the reported frequent comorbidities of IBS with other chronic pain and psychiatric conditions it is becoming increasingly recognized that IBS is part of a constellation of symptoms that occur on a larger spectrum of altered brain-body interactions [134, 135]. This concept is consistent with the “somatic symptom disorder” concept, previously proposed [2]. The main co-occurring symptoms include hypersensitivity to multiple internal and external sensory stimuli, which could explain the observed association with a variety of seemingly unrelated external and internal factors, previously reported. Other co-occurring symptoms include mood problems, fatigue, and problems with sleep onset and maintenance, as well as memory disturbance [134]. The neurogenetic basis integrating mood/anxiety and central amplification of sensory inputs (“central sensitization”) based on many of these genetic hits have been well established, which will be discussed below.

Known functions of *NCAM1*, *DOCK9*, and *PHF2* and possible roles in IBS pathophysiology are summarized in Table 2.

**Table 2:** Genetic Factors in the Neurobiology of IBS.

Gene	Known/Implicated Functions	Supporting neurological findings in IBS
*NCAM1*	• Implicated in mood/psychiatric disorders such as neuroticism [221]. • Implicated in central sensitization [222]. • Upregulated rapid turnover of *NCAM1* in the ACC contributes to central sensitization by increasing neuronal connectivity [222].	• The ACC is a main hub of the salience network found to be highly implicated in IBS pathophysiology [33, 53]. • Reduction in ACC connectivity and increased positive mood in response to cognitive behavioral therapy was associated with IBS symptom improvement [177].
*DOCK9*	• Implicated in proper growth and development of hippocampal neurons [131].	• Increased functional connectivity of the hippocampus observed in IBS [223]. • Rodent models show early life trauma can lead to increased expression of hippocampal AMPA GluR2 receptors and associated visceral hypersensitivity. AMPA GluR2 inhibitors alleviated visceral hypersensitivity [224]. • Lower volumes of the hippocampus have been observed in patients with IBS [117]. • Greater integrity of the inferior longitudinal fasciculus predicts symptom improvement over 3–12 months [109]. Analogous to rodent models, the integrity of this bundle is affected by early life trauma [225].
*PHF2*	• Essential for hippocampal-dependent learning and memory. Facilitates LTP in CA1 pyramidal neurons [226].	• Hippocampal CA1 LTP is associated with increased nociceptive processing and anxiety-induced hyperalgesia [227, 228]. • Administration of a CRF-R1 antagonist has been shown to reduce upregulated CRF via suppressed BOLD activity in the hippocampus during conditioned fear extinction [170, 171].
*CADM2*	• Encodes a member of the synaptic cell adhesion molecule 1 (SynCAM) family involved in synaptic organization and signaling, more abundantly expressed in brain tissue compared to other tissue [69, 229].	• Mechanistic studies needed in the context of IBS
*BAG6*	• Involved in membrane protein quality control, apoptosis, gene regulation, and immunoregulation [230–232].	• Mechanistic studies needed in the context of IBS
*CKAP2*	• Encodes a microtubule-associated protein playing a large role in cell division during mitosis and cell proliferation [233, 234].	• Mechanistic studies needed in the context of IBS

### Central sensitization and comorbid chronic pain conditions

The primary mechanism for the core symptom of persistent, chronically recurring abdominal pain that patients with IBS report is thought to result from alterations in the central processing of sensory input from the gut, also referred to as central sensitization [134, 136]. The term was originally coined to represent the specific spinal mechanisms responsible for the amplification of nociceptive signaling involving spinal activation of the NMDA receptor [137, 138], and is present in various chronic pain disorders such as chronic neuropathic pain, fibromyalgia, headaches, and IBS [6, 134, 139–141]. Today, it is understood that spinal and supraspinal mechanisms both play key roles in the development and maintenance of central sensitization. Based on rodent models of pain, plausible spinal mechanisms include alterations in converging sensory input from different sites on the GI tract and body, temporal and spatial summation, reduced endogenous dorsal horn inhibition, and glial cell activation. Based on human brain imaging studies, supraspinal mechanisms include an altered balance between facilitatory and inhibitory endogenous pain modulation influences, hyperconnectivity between brain networks, alterations of gray matter architecture, elevated CSF glutamate and substance P levels, reduced GABAergic transmission, altered noradrenergic signaling/receptors, and glial cell activation [122, 134].

The large overlap - up to a 4.27 odds ratio - between psychiatric phenotypes (primarily anxiety and depression [136, 142]) and IBS and other chronic pain disorders, as well as genetic overlap [8, 143–145] mentioned earlier, suggests central sensitization as a possible shared pathophysiological factor (p factor) [134, 146–148]. The concept of central sensitization was introduced in psychological research in the 1990s based on the observation that highly sensitive persons (HSPs) often share a history of early adversity, psychological profile of introversion (“neuroticism”), and greater emotionality [149]. Patients with IBS are significantly more likely to exhibit qualities of HSPs, and show central sensitization which is expressed as general sensory hypersensitivity [150]. The association between chronic pain disorders, psychiatric symptoms, and mechanisms of central sensitization is likely due to the above-mentioned supraspinal alterations, including monoamine neurotransmitter systems (i.e., serotonin, dopamine, noradrenaline), the amino acid GABA, and brain regions underlying both pain transmission/modulation and mood disorders [151, 152]. Striato-thalamic-frontal cortical pathways including the prefrontal cortex, amygdala, nucleus accumbens, and thalamic nuclei are key hubs, and alterations in neuronal firing and communication underlie sensory sensitivity and psychiatric symptoms including altered perception, arousal, cognition, and mood [152–154]. Behaviorally, chronification of central sensitization and negative mood states have been proposed to be in the same continuum of aversion, such that pain motivates the avoidance of further injury, and anxiety promotes behaviors that diminish anticipated danger [154].

An extensive literature supports the importance of early programming by early adverse life (EAL) events for the development not only of IBS [76], but also of other chronic pain conditions and psychiatric syndromes [155, 156]. Perturbations to the developing brain play a large sole in sensitizing cortical nociceptive circuitry [157], with the most mechanistic study in humans showing larger event-related potentials (ERPs) to nociceptive stimuli, but not tactical stimuli in infants exposed to many invasive, skin-breaking, painful procedures and morphine [158]. Moreover, up to 68.4% of children who are exposed to early life traumatic events such as the NICU can develop chronic pain by age 10. Greater amounts of pain-related stressors, painful procedures, and morphine are associated with lower global gray matter volumes throughout childhood [159, 160]. In addition to the well documented changes in stress response systems [161–163], the effect of early-life dietary influences on the gut microbiome and the BGM axis have received increasing attention, even though a direct link with chronic abdominal pain has not been established [164, 165].

### Clinical and therapeutic implications

Despite a decades-long effort by the pharmaceutical industry, a large number of IBS candidate drugs identified and validated in preclinical models and targeted at both central and gut mechanisms have failed, either due to lack of efficacy or serious side effects [16]. Of the small number of new drugs obtaining FDA approval, efficacy above placebo has generally not exceeded 10% in phase 3 trials. The great majority of available, FDA approved IBS medications are targeted at intestinal secretion and motility, and the gut microbiome with the goal to improve altered bowel habits and bloating-type symptoms in subgroups of patients [16].

Pharmacological treatments have been clinically divided into first and second-line approaches [16], and are aimed at specific symptoms. Moderate quality data has shown low-dose tricyclic antidepressants and SSRIs to be effective for pain (primarily the former) and comorbid anxiety and depression (primarily the latter) [16, 18]. As 5-HT receptor-mediated signaling plays important roles both in the brain, as well as in the gut, there is a good rationale for IBS treatments targeted at these receptors. 5-HT released from enterochromaffin cells mediates many GI functions including peristalsis, secretion, pain, and nausea via receptors on ENS and vagal nerve endings [31]. For example, 5HT-3 receptor antagonists (acting on both gut and brain-located 5HT-3 receptors (such as alosteron, and ramosteron) have shown effectiveness in slowing colonic transit, improving diarrhea, and reducing visceral pain in well-designed randomized controlled trials [16]. High-quality preclinical data has shown the antagonism of 5HT-3 receptors on the area postrema and vagus nerve have shown a reduction of visceral pain and diarrhea [16, 18, 166], and older data have demonstrated anxiolytic effects [167–169].

Despite evidence obtained in rodent models of IBS, efforts to develop peripheral visceral analgesics or central stress modulators (antagonists for CRF-1 and NK-1 receptors) have failed to show therapeutic benefits in IBS. This is surprising, as multiple preclinical studies as well as a human brain imaging study had demonstrated effectiveness of the CRF-R1 antagonist Emicerfont (GW876008) on evoked visceral pain and on central stress circuits [170, 171]. Because of these disappointing results, increased attention has been shifted to behavioral treatments, including gut-directed hypnosis [21, 81, 172–175], mindfulness-based stress reduction [176], and cognitive behavioral approaches [19, 20, 177–179]. Several of these therapeutic approaches have shown promise in improving IBS symptoms, and a few studies have demonstrated associated neurobiological effects on brain mechanisms in salience, emotional arousal, and executive networks [172, 177].

As access to therapists specialized in these behavioral IBS treatments is limited, and traditional delivery is time-consuming, web-based versions of these therapies have been evaluated, some of which have been FDA approved and are becoming available to patients [180]. In addition, several randomized controlled studies have shown some benefits of certain dietary interventions (low FODMAP diet [16]), and microbiome-targeted treatments (probiotics, antibiotics) [181].

## Summary and conclusions

Even though in subsets of patients, SSRIs and bowel movement targeted therapies are helpful, the model of IBS presented in this review provides precedence for a multidisciplinary therapeutic approach including pharmacological, behavioral, and dietary approaches. Current evidence suggests that there are significant interindividual variations in the response to such therapies, including the predominant bowel habit subtype, severity of gut and psychiatric symptoms, and possibly the presence of gut microbial alterations.

There is growing evidence from clinical, preclinical, and genetic studies supporting the existence of shared p factors in IBS and often comorbid gastrointestinal and non-gastrointestinal pain conditions, as well as psychiatric conditions. Despite shared vulnerability genes, different influences from the environment (exposome) in particular during childhood ultimately shape the specific clinical phenotype. The emerging disease model can explain the failure of reductionistic single mechanism targeted treatment approaches, and is consistent with the evidence for the effectiveness of personalized multidisciplinary approaches involving behavioral, dietary, and pharmacological interventions.

## Glossary

irritable bowel syndrome (IBS); brain-gut-microbiome (BGM); gastrointestinal (GI); enteric nervous system (ENS); central nervous system (CNS); synaptic cell adhesion molecules (SynCAMs); default mode network (DMN); salience network (SAL); sensorimotornNetwork (SMN); central autonomic network (CAN); central executive network (CEN); locus coeruleus (LC); periaqueductal grey (PAG); dorsal anterior cingulate cortex (dACC); posterior cingulate cortex (PCC); N-methyl-D-aspartate (NMDA); gamma-aminobutyric acid (GABA); cerebrospinal fluid (CSF); early adverse life events (EAL); serotonin (5-HT); selective serotonin reuptake inhibitor (SSRI); long-term potentiation (LTP); event-related potentials (ERPs).
